# Pityriasis lichenoides et varioliformis acuta in a patient treated with cevostamab

**DOI:** 10.1016/j.jdcr.2024.02.021

**Published:** 2024-03-05

**Authors:** Jeremy Orloff, Dev D. Patel, Camille M. Powers, Austin J. Piontkowski, Robert G. Phelps, Joshua Richter, Nicholas Gulati

**Affiliations:** a Department of Dermatology, Icahn School of Medicine at Mount Sinai, New York, New York; b Departments of Dermatology and Pathology, Icahn School of Medicine at Mount Sinai, New York, New York; c Division of Hematology and Medical Oncology, Tisch Cancer Institute, Icahn School of Medicine at Mount Sinai, New York, New York

**Keywords:** Bi-specific antibody, BsAb, disorders, drug reactions, immunomodulation, immunotherapy, lymphoproliferative, multiple myeloma, oncoderm, pathology, PLC, PLEVA, pityriasis lichenoides et varioliformis acuta

## Introduction

Multiple myeloma (MM) is a hematological malignancy caused by clonal expansion of neoplastic plasma cells. The disease is characterized by paraprotein, anemia, renal dysfunction, bone damage, and immunosuppression.^1^ Bispecific antibodies (BsAbs), which redirect T cells to kill tumor cells, represent a new class of therapeutics for MM cases that are refractory to standard treatments. Cevostamab is an experimental BsAb which simultaneously targets the cluster of differentiation (CD)3 T-cell receptor and Fc receptor-like protein 5, a B-cell marker with increased expression on MM cells.^2^

We present a case of a patient with MM, who participated in a phase 1 clinical trial of cevostamab for patients with relapsed/refractory MM, who developed pityriasis lichenoides et varioliformis acuta (PLEVA).

## Case

A 79-year-old man with a history of plaque psoriasis and refractory MM presented with a non-painful, non-pruritic rash on his extremities. He had been first diagnosed with pancytopenia 5 years prior. His medication history included long-term doxycycline use for Lyme disease and ustekinumab for psoriasis stopped 11 and 5 years prior to presentation, respectively. The patient’s psoriasis had been well controlled without further treatment. Comorbidities included hypertension, coronary artery disease, and benign prostatic hyperplasia. His daily medications included acyclovir, apixaban, atorvastatin, finasteride, metoprolol, omeprazole, and tamsulosin, all of which were started at least 4 months prior to presentation. The patient first reported the rash approximately 16 weeks after his first loading dose (approximately 12 weeks after his first full dose) and 3 weeks after his last infusion of cevostamab. His trial participation was paused pending a dermatologic evaluation.

**Fig 1:**
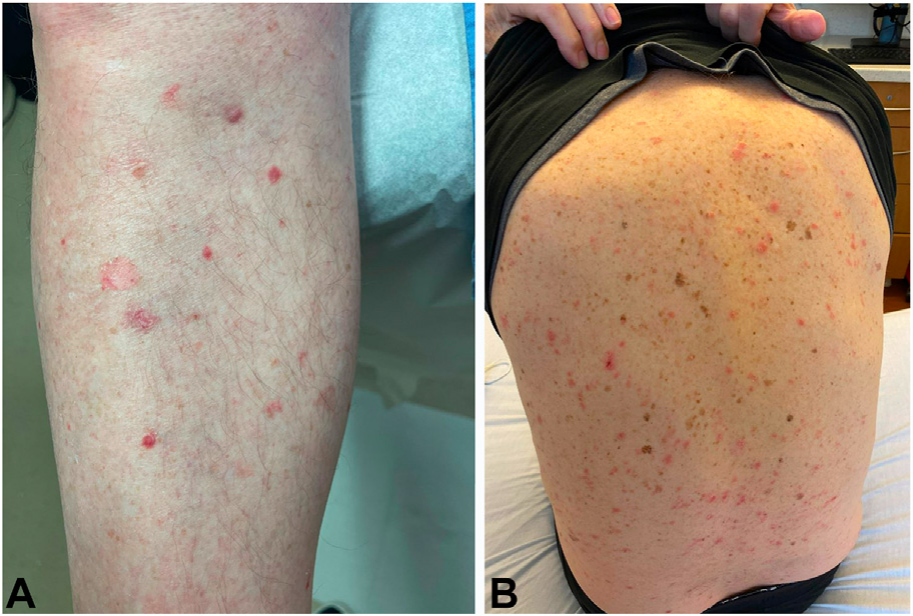
Clinical images of guttate erythematous papules and sharply demarcated macules, some with central scaling on (**A**) the left lower leg and (**B**) the back.

**Fig 2:**
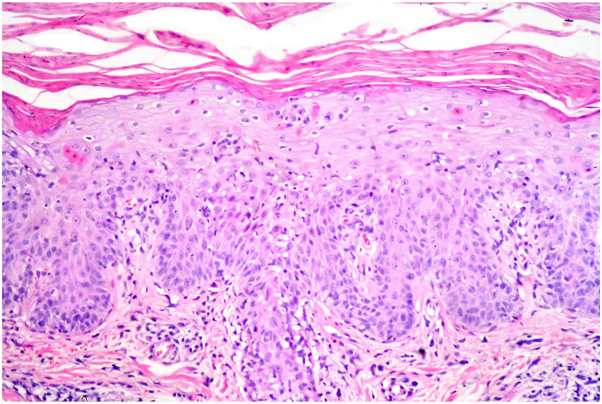
Histopathology of lesion on left thigh. An acanthotic epidermis with scattered necrotic keratinocytes shows a confluent neutrophil rich parakeratosis. The dermis contains a mononuclear infiltrate showing extensive exocytosis into the epidermis (Hematoxylin-eosin, 20×).

Physical examination revealed scattered guttate erythematous papules and sharply demarcated macules, some with central scaling, on the arms, legs, and back (Fig 1). Histology revealed that the superficial dermis contained a dense lichenoid mononuclear infiltrate with exocytosis into the epidermis. The epidermis exhibited pronounced hydropic change and foci of keratinocyte necrosis. Scattered extravasated erythrocytes were also present (Fig 2). Based on these findings, the patient was diagnosed with PLEVA.

The patient was prescribed topical triamcinolone but reported not using it 3 weeks later. The rash expanded, and his next cevostamab infusion was deferred given his cutaneous eruption. He was prescribed a 6-day tapered course of methylprednisolone, starting at 24 mg/d. Despite this treatment, his rash continued to worsen. Given that it covered >30% body surface area, the patient was deemed to have a persistent grade 3 adverse event. He elected to withdraw from the clinical trial and reported that the rash was slowly improving 3 months after discontinuation of the experimental therapy.

## Discussion

PLEVA is a rare acute disease characterized by scattered round erythematous macules and papules. As these lesions evolve, they form a scale which detaches at the periphery and may form a central punctum and ulcerate. As in this case, the presentation of PLEVA can resemble guttate psoriasis.^3^ The natural history of the disease generally involves flares over the course of several weeks with eventual spontaneous resolution. In some cases, patients may progress to the chronic form of the disease, pityriasis lichenoides chronica (PLC).

Though there is some evidence that PLEVA represents a cutaneous immune response to an infection-associated vasculitis or immune-complex mediated vasculitis, multiple studies have shown that PLEVA is a T-cell lymphoproliferative process. On histopathology, PLEVA demonstrates a T-cell dominant infiltrate,^4^ and a dominant T-cell clone has been found in a majority of cases.^3^ Magro et al^5^ argued that PLEVA and PLC represent a form of T-cell dyscrasia based on the reduced expression of CD7 and CD62L, along with the presence of clonality. They also cited cases which evolved into mycosis fungoides as evidence of an underlying T-cell process.

A recent case described PLC in a 69-year-old man treated with an anti-CD19 therapy for diffuse large B-cell lymphoma. In that case, the authors posited that T-cell dysregulation due to altered B-cell response could explain the development of PLC.^6^ In our case, the patient received a treatment specifically designed to alter T-cell behavior, which may align with this proposed mechanism of pathogenesis.

To the authors’ knowledge, this is the first reported case of PLEVA in a patient undergoing treatment with a BsAb. BsAbs bind a T-cell as well as a tumor cell, inducing the T-cell to mount an immune response against the tumor cells. In pharmacodynamic studies of cevostamab, not only was T-cell activation noted, but T-cell proliferation was also observed in responsive patients.^7^

CD3 BsAbs have only previously been associated with nonspecific rashes. Chari et al^8^ reported 3 dose-limiting grade 3 maculopapular rashes in patients on talquetamab, which targets G protein-coupled receptor class C group 5 member D on MM cells. Maculopapular rash was similarly the most common grade 3 or higher adverse event for patients on tebentafusp, a BsAb CD3 T-cell engager approved for uveal melanoma.^9^

The T-cell proliferation known to be caused by BsAbs is in line with our understanding of the likely pathogenesis of PLEVA and the modest improvement reported in the absence of treatment suggests a possible causal relationship in this case. However, it should be noted that PLEVA can occur sporadically without an identifiable cause, and that PLC has been described in patients with MM treated with other agents.^10^

## Conclusion

Although PLEVA is benign, responsive to topical therapies, and generally self-resolving, severe and diffuse cases may significantly impact a patient’s quality of life and interfere with life-saving treatments, as evidenced by this patient discontinuing his clinical trial. As BsAbs are a new and increasingly used class of therapeutics, dermatologists must remain vigilant for cutaneous complications.

## Conflicts of interest

Dr Richter reports research funding and consulting fees from Genentech. Authors Orloff, Patel, Powers, Piontkowski, Phelps, and Gulati have no disclosures.
